# Epigenetic Control of Phenotypic Plasticity in the Filamentous Fungus *Neurospora crassa*

**DOI:** 10.1534/g3.116.033860

**Published:** 2016-09-29

**Authors:** Ilkka Kronholm, Hanna Johannesson, Tarmo Ketola

**Affiliations:** * Centre of Excellence in Biological Interactions, Department of Biological and Environmental Sciences, University of Jyväskylä, FI-40014, Finland; † Department of Organismal Biology, University of Uppsala, 752 36, Sweden

**Keywords:** reaction norm, DNA methylation, histone methylation, histone deacetylation, RNA interference, fungi

## Abstract

Phenotypic plasticity is the ability of a genotype to produce different phenotypes under different environmental or developmental conditions. Phenotypic plasticity is a ubiquitous feature of living organisms, and is typically based on variable patterns of gene expression. However, the mechanisms by which gene expression is influenced and regulated during plastic responses are poorly understood in most organisms. While modifications to DNA and histone proteins have been implicated as likely candidates for generating and regulating phenotypic plasticity, specific details of each modification and its mode of operation have remained largely unknown. In this study, we investigated how epigenetic mechanisms affect phenotypic plasticity in the filamentous fungus *Neurospora crassa*. By measuring reaction norms of strains that are deficient in one of several key physiological processes, we show that epigenetic mechanisms play a role in homeostasis and phenotypic plasticity of the fungus across a range of controlled environments. In general, effects on plasticity are specific to an environment and mechanism, indicating that epigenetic regulation is context dependent and is not governed by general plasticity genes. Specifically, we found that, in *Neurospora*, histone methylation at H3K36 affected plastic response to high temperatures, H3K4 methylation affected plastic response to pH, but H3K27 methylation had no effect. Similarly, DNA methylation had only a small effect in response to sucrose. Histone deacetylation mainly decreased reaction norm elevation, as did genes involved in histone demethylation and acetylation. In contrast, the RNA interference pathway was involved in plastic responses to multiple environments.

Natural environments are in a constant state of change. Organisms must cope with different environments and their dynamism by adjusting their development, behavior, and reproduction, while always maintaining physiological homeostasis. Phenotypic plasticity is defined as the ability of an organism to express different phenotypes in response to environmental changes (DeWitt and Scheiner 2004), where phenotype can be any morphological, physiological, or behavioral trait. In most cases, phenotypic plasticity is based on adjusting the patterns of gene expression (Nicotra *et al.* 2010). Phenotypic plasticity plays an important role in buffering fitness across a range of environments, and can help evolutionary adaptation to extreme environments (Lande 2009; Chevin *et al.* 2010; Draghi and Whitlock 2012). Specifically, plasticity can facilitate adaptation by increasing population size in the novel environment (Chevin *et al.* 2010), and by generating phenotypic variation that can be selected if it is heritable (Pál 1998). Heritable phenotypic variation could potentially be achieved with different mechanisms, one of them being plasticity mediated by epigenetic mechanisms. Interestingly, evolutionary models incorporating heritable genetic and epigenetic systems suggest that the latter play a significant role in adaptation (Day and Bonduriansky 2011; Klironomos *et al.* 2013; Kronholm and Collins 2016).

Transcriptomic studies have shown that environment has a large effect on gene expression profiles (Gibson 2008; Nicotra *et al.* 2010; Alvarez *et al.* 2015). The classical view is that those patterns are regulated by transcriptional activator and repressor proteins (Davidson 2006). However, there is increasing interest in the influence of epigenetic changes (*e.g.*, DNA methylation and histone modifications) on gene expression (de la Paz Sanchez *et al.* 2015). Furthermore, epigenetic mechanisms are involved in phenotypic plasticity (Slepecky and Starmer 2009; Bossdorf *et al.* 2010; Herrera *et al.* 2012; Baerwald *et al.* 2016) and transgenerational inheritance (Verhoeven *et al.* 2010; Verhoeven and van Gurp 2012; Luna and Ton 2012; Rasmann *et al.* 2012; Ou *et al.* 2012; Öst *et al.* 2014; Siklenka *et al.* 2015). While experiments that have artificially induced variation in DNA methylation have shown how this factor can contribute significantly to phenotypic variation (Cortijo *et al.* 2014) and plasticity (Kooke *et al.* 2015), the relative importance of other epigenetic modifications—even the extent to which their effects are environmentally dependent—remains unclear.

The filamentous fungus *Neurospora crassa* is a good model system with which to study epigenetics as it has nearly all the epigenetic mechanisms that higher eukaryotes have, but many of these mechanisms are not essential for viability (Allshire and Selker 2009). It is also easy to do controlled experiments in different environments as the fungus can be propagated asexually. As phenotypic plasticity in fungi has not been studied extensively (Slepecky and Starmer 2009), we need to first establish how *N. crassa* responds to different environments phenotypically, and ask what is the role of epigenetic mechanisms? This is necessary in order to study the possible transgenerational effects or the role of plasticity in adaptation in the future.

To explore the extent to which various epigenetic mechanisms are involved in phenotypic plasticity, we used an experimental set of 25 different mutant strains of *N. crassa*, each of which is deficient in a particular chromatin modification, affecting: DNA methylation (three mutants), histone methylation (five), histone deacetylation (eight), histone acetylation (two), histone demethylation (two), and RNA interference (five). We selected mutants that either had been previously characterized and were known to affect different epigenetic modifications, or based on their homology to genes known to modify chromatin in other organisms.

We measured the reaction norms—the phenotypic expression of a trait for a genotype across a range of environments—of each strain with respect to four different environmental variables: temperature, osmotic stress (NaCl), sucrose concentration, and pH, to investigate the potential effects of each epigenetic mechanism on phenotypic plasticity across a range of environments. A reaction norm is visualized by plotting the measurable performance (*e.g.*, mycelial growth rate) of an organism scored at different values of an environmental parameter. Reaction norms can be described by their shape and elevation; shape refers to variation in phenotype that contributes to genotype by environment interaction, and elevation means variation in phenotype that contributes to the genotypic effect only. If epigenetic modifications play a role in phenotypic plasticity, we expect to see differences in reaction norm shapes for the mutant strain and wild type. Differences in reaction norm elevations indicate that the epigenetic modification is required for normal cellular function rather than a physiologically plastic response.

We show that epigenetic mechanisms are involved in plastic responses of *N. crassa*. These responses involve a specific epigenetic modification in a particular environment, *e.g.*, histone modifications are important in the response to temperature and pH, and the RNA interference pathway also has notable effects. In contrast, lacking the ability to carry out DNA methylation had little effect on strain performance in any of the trial environments.

## Materials and Methods

### Neurospora strains

We used 25 different strains from the *N. crassa* knockout collection (Colot *et al.* 2006) to investigate the role of epigenetic mechanisms in phenotypic plasticity. The mutants were generated by replacing the entire open reading frame of the target gene with an *hph* cassette, which confers resistance to the antibiotic Hygromycin B. Strains were obtained from the Fungal Genetics Stock Center (FGSC) (McCluskey *et al.* 2010); Supplemental Material, Table S1 shows the strains used in this study, and Table 1 shows the genotypes of those used in the experiments. We included strains that are viable in the homokaryotic state and for which we could confirm the gene deletion. Strain FGSC # 4200 was used as a wild-type control for the reaction norm measurements. We grouped the strains into five categories based on the epigenetic mechanism in which they are deficient: DNA methylation, histone methylation, histone deacetylation, RNA interference, and “other,” which included two putative histone demethylases and two histone acetyl transferases (Table 1).

**Table 1:** Mutant strains used in this study

Gene	Gene ID	Epigenetic Mechanism	Modification	Function
*dim-2*	NCU02247	DNA methylation	DNA me	DNA methyltransferase
*dmm-1*	NCU01554	DNA methylation	DNA me	Controls the spreading of DNA methylation from heterochromatic regions
*dmm-2*	NCU08289	DNA methylation	DNA me	Controls the spreading of DNA methylation from heterochromatic regions
*dim-5*	NCU04402	Histone methylation	H3K9me3	H3-specific methyltransferase; H3K9 is a mark for silent heterochromatin, guides DNA methylation
*set-1*	NCU01206	Histone methylation	H3K4me3	H3-specific methyltransferase; H3K4 trimethylation affected
*set-2*	NCU00269	Histone methylation	H3K36me	H3-specific methyltransferase; H3K36; needed for correct transcriptional elongation
*set-7*	NCU07496	Histone methylation	H3K27me3	H3-specific methyltransferase; H3K27; catalytic subunit of PRC2
*npf*	NCU06679	Histone methylation	H3K27me3	Homolog of Drosophila p55; part of chromatin remodeling complex
*nst-1*	NCU04737	Histone deacetylation	H4AcK16	Histone deacetylase (Class III); deacetylates H4K16, mutation causes activation of a silenced transgene
*nst-2*	NCU00523	Histone deacetylation	Unknown	Inferred HDAC; mutation causes activation of a silenced transgene
*nst-4*	NCU04859	Histone deacetylation	Unknown	Inferred HDAC; mutation causes activation of a silenced transgene
*nst-6*	NCU05973	Histone deacetylation	Unknown	Inferred HDAC; mutation causes activation of a silenced transgene
*nst-7*	NCU07624	Histone deacetylation	Unknown	Inferred HDAC; mutation causes activation of a silenced transgene
*hda-1*	NCU01525	Histone deacetylation	H2B	Histone deacetylase (Class I); Homolog of yeast *Hda1*; partial loss of DNA methylation, increased acetylation
*hda-2*	NCU02795	Histone deacetylation	Unknown	Inferred HDAC; homolog of yeast *Hos2*
*hda-4*	NCU07018	Histone deacetylation	Unknown	Inferred HDAC; homolog of yeast *Hos3*; increased acetylation at all sites (except H3K19)
*qde-1*	NCU07534	RNA interference	NA	RNA- and DNA-dependent RNA polymerase; initiation of the RNAi pathway
*qde-2*	NCU04730	RNA interference	NA	Argonaute; mutations abolish milRNA processing ability
*dcl-1*	NCU08270	RNA interference	NA	Dicer ribonuclease; maturation of milRNAs
*dcl-2*	NCU06766	RNA interference	NA	Dicer ribonuclease; maturation of milRNAs
*qip*	NCU00076	RNA interference	NA	Exonuclease; milRNA maturation defective
*aof2*	NCU09120	Other	Unknown	Inferred histone demethylase; homolog of *S. pombe lsd1* and *Aspergillus HdmA*
*elp3*	NCU01229	Other	Unknown	Inferred histone acetyl transferase
*lid2*	NCU03505	Other	Unknown	Inferred histone H3K4 demethylase; homolog of *S. pombe lid2*
*ngf-1*	NCU10847	Other	H3AcK14	Histone acetyl transferase; acetylation of H3K14

Gene ID is based on the *N. crassa* genome assembly NC12. Epigenetic mechanism is the classification for the strains studied here. Modification is the chromatin modification affected by the mutation, and function describes what is known about the biochemical activity of the protein. NA, not applicable; HDAC, histone deacetylase; milRNA, microRNA-like RNA.

### Genotyping

We confirmed that the mutant strains indeed have deletions, and verified their mating types by PCR. We relied on a rapid method of DNA extraction from conidia or asexual spores (Henderson *et al.* 2005). We grew a strain in an agar slant for 3–5 d until orange-colored spores were visible. Spores were then collected and suspended in water containing 0.01% Tween-80. Conidial boiling buffer was prepared by combining 100 parts of 50 mM Tris pH 8 and two parts of 0.5 M EDTA pH 8.5. We then distributed 10 μl of conidial boiling buffer across a 96-well PCR plate, and combined 40 μl of conidial suspension to each well. The loaded plate was boiled for 10 min at 98° in a thermal cycler, and 2 μl of the resulting suspension was used as template in subsequent PCR reactions.

A strain’s mating type was determined by PCR in a single 10 μl reaction containing four primers, *i.e.*, two pairs. These two primer pairs (Table S2) were designed using the *N. crassa* genome sequence to amplify a 200-bp fragment from the *mat a* locus, and a 400-bp fragment from the *mat A* locus. PCR products were resolved by electrophoresis on a 2–3% agarose gel. To confirm that our mutant strains indeed have deletions, we designed primers for each of the target genes that amplified a fragment of ∼500-bp, and checked the absence of the deleted genes by PCR. We set up PCR reactions with the high-fidelity Phusion DNA polymerase (Thermo Scientific) according to the manufacturer’s instructions: the final PCR reactions contained 1× Phusion HF buffer, 0.2 mM each dNTP, 0.5 μM each primer, and 0.2 or 0.4 units of Phusion DNA polymerase for 10 or 20 μl reactions, respectively. All PCR reactions were run with the following thermal profile: initial denaturation of 98° for 30 sec, then 35 cycles of 98° for 5 sec, variable annealing for 10 sec, extension at 72°C for 20 sec, and a final extension at 72° for 1 min. Primer sequences and their annealing temperatures are given in Table S2.

### Growth measurements

In general, standard laboratory protocols for *Neurospora* (Davis and de Serres 1970) were followed. We grew *N. crassa* on Vogel’s growth medium (Metzenberg 2003) with 1.5% agar appropriately supplemented for the different environments listed below. To measure growth rate of the strains, we used the race tube method of Ryan *et al.* (1943), with tubes prepared following White and Woodward (1995). Briefly, we filled 25 ml plastic serological pipettes (Sarsted) with 10 ml of molten agar and placed them horizontally so that the agar solidified at the bottom of the pipettes. The tip of the pipette was snapped off, and that end was inoculated with conidia and subsequently sealed with Parafilm. The other end of the pipette contained a cotton plug. Growth of the mycelial front in the tube was measured by marking its position twice a day (every 8th and 16th hr). Growth was followed typically for a period of 104 hr but up to 152 hr in the osmotic stress environment.

To estimate growth rates, we used a simple linear regression of time against the distance the mycelial front had grown in a race tube. The first marking was made when the mycelial front was clearly visible, and growth rate data were collected from this point on. Growth immediately following inoculation until the mycelial front was first visible was not included. This effectively corrects for possible differences in initial growth rate due to inoculum size. We extracted the slope of the regression line for each growth assay to obtain the mycelial growth rate as mm/hr. Growth rates were used as a dependent variable in subsequent analyses.

### Reaction norms

As a measure of phenotypic plasticity, we measured reaction norms of the different strains with respect to four different environmental parameters: temperature, osmotic stress (NaCl), sucrose concentration, and pH. We used six different settings within each parameter, 26 different genotypes with five replicate growth rate measurements in each treatment combination, yielding 3120 growth rate measurements in total. The different parameter settings were: 15, 20, 25, 30, 35, and 40° for the temperature; 0, 0.2, 0.4, 0.8, 1.2, and 1.6 M of NaCl added for osmotic stress; 0.015, 0.15, 1.5, 5, 15, and 30% (w/v) sucrose added for sucrose concentration; or pH adjusted to 4.0, 5.0, 5.8, 7.0, 8.0, and 9.0. Except for temperature, which was controlled by the growth chamber, the standard growth medium was manipulated by either adding NaCl, varying the sucrose level, or adjusting pH with either HCl or NaOH. We did not control for any changes in nutrient availability in the medium that may result from pH changes, and allowed the environmental changes to be complex.

Normal growth conditions were 25°, 0 M NaCl, 1.5% sucrose, pH 5.8, and constant darkness. Independent measurements were collected during all experiments under these “control” conditions. The reaction norm experiment was performed in growth chambers, and replicate measurements were blocked in time by replicates, such that each strain and environmental setting ran simultaneously, and the growth tubes were randomized in the growth chamber. However, since the temperature treatment had to be applied to the entire growth chamber, for the temperature treatment, we used two growth chambers of the same model (Lab companion ILP-02/12; Jeio Tech, South Korea), where we always switched the identity of the growth chamber between replicate measurements. For example, for replicate 1, chamber A was set to 25° and chamber B to 30°; for replicate 2, chamber A was set to 30° and chamber B to 25°; and so on. This allowed us to check for any possible effect of either growth chamber independent of temperature.

### Backcrossing and validation

To control for possible genetic background effects between the mutants and the control strain 4200, we performed a validation experiment with strains where we had backcrossed the mutant strain five times into FGSC # 2489 background using standard crossing techniques (Davis and de Serres 1970). The pedigree of the wild type strains 4200 and 2489 is known (Mylyk *et al.* 1974; Newmayer *et al.* 1987; Perkins 2004). They are nearly isogenic, expect for the mating type locus, and the theoretical expectation from the number of backcrosses is that they share >99.99% of their genetic background. The deletion mutants were generated by transforming the strain 4200 (Colot *et al.* 2006). Theoretically, after five backcrosses, the backcrossed strains should share 98.44% of their genetic background with 2489, but as 4200 and 2489 were already nearly isogenic, they share more of their genetic background than would be expected from five backcrosses.

The sexual cycle can be induced by growing *N. crassa* in a low-nitrogen medium. The fungus has two different mating types: *mat A* and *mat a*. When the two types meet, a conidial or a mycelial cell of one type fertilizes the protoperitechium, the “female” organ, of the other type, and the different nuclei fuse and undergo meiosis to yield haploid spores. We used crossing medium (Davis and de Serres 1970) containing 0.2% sucrose, but, instead of agar, we used 5 ml of liquid medium in a large 20 × 150 mm test tube with a 40 × 90 mm piece of vertically folded filter paper that wicks the medium by capillary action. The filter paper was inoculated with conidia from the opposite mating types. We germinated ascospores on plates with sorbose to induce colonial growth and 200 μg/ml of Hygromycin B, where appropriate, to select for mutant-strain progeny. We checked mating type of the progeny, and confirmed the gene deletions by PCR in each round of backcrossing as above. As 2489 is *mat A*, we selected *mat a* progeny until a final backcross generation was recovered containing mutant genotypes of both mating types.

Based on the results of the reaction norm experiment, we selected strains *dim-2*, *dmm-2*, *hda-1*, *qde-2*, *qip*, *aof2*, *lid2*, and *set-7* for validation experiments with the backcrossed strains. For temperature measurements, we measured *dim-2*, *qip*, *set-7*, and *aof2* in 40°, *qde-2*, *lid2*, and *aof2* in 35 and 30°. For the pH environment, we measured *dmm-2* at pH 4, and *dmm-2* and *qde-2* at pH 9. In osmotic stress, we measured only *hda-1* at 0.8 M NaCl. For sucrose concentration, we measured *dim-2*, *qde-2*, and *set-7* at 30% sucrose, and *qde-2* at 0.015% sucrose. Growth rate was measured as in the reaction norm experiment, and assays were replicated 12 times in the validation experiment, each including strain 2489 as a control.

### Data analysis

We used an ANOVA to investigate whether different epigenetic mechanisms have different effects, and whether they are specific to particular environments. Because the reaction norms were nonlinear, we encoded the different parameter settings as factors. This allowed us to analyze all of the data together despite differences in reaction norm shape. We fitted a mixed model using the “lmer” function in R (R Core Team 2013) with tests performed using the “lmerTest” package (Kuznetsova *et al.* 2015). This package implements *F*-tests using type III sums of squares with Satterthwaite correction for degrees of freedom. Type III sums of squares were used to interpret results according to the elevation and shape of the reaction norms, following the phenotypic plasticity literature. The model was

$$y_{ijkl}=\mu +M_{i}+S_{j}+E_{k\left(j\right)}+G_{l\left(i\right)}+M_{i}\times S_{j}+M_{i}\times E_{k\left(j\right)}+S_{j}\times G_{l\left(i\right)}$$ (1)

where *μ* is the intercept, $M_{i}$ is the *i*th epigenetic mechanism, $S_{j}$ is the *j*th environmental parameter (temperature, salt, sucrose, or pH stress), $E_{k\left(j\right)}$ is the *k*th parameter setting nested within parameter *j*, and $G_{l\left(i\right)}$ is the *l*th genotype nested within epigenetic mechanism *i*. Epigenetic mechanism, environmental parameter, and parameter setting were fitted as fixed factors, while genotype was fitted as a random factor. We subsequently analyzed each of the different environmental parameters separately with the model

$$y_{ikl}=\mu +M_{i}+E_{k}+G_{l\left(i\right)}+M_{i}\times E_{k}+E_{k}\times G_{l\left(i\right)}$$ (2)

with terms the same as above. We also performed a pairwise ANOVA, comparing the control to each of the mutants in each of the four different environments. This allowed us to test whether the genotype × environmental level interaction term was significant, reflecting changes in reaction norm shape. We adjusted P-values for multiple testing using the Bonferroni-Holm correction (Holm 1979).

### Estimating reaction norm optima

To characterize reaction norm optima for the different strains, we fitted natural splines, *i.e.*, functions built piecemeal from polynomial functions (Venables and Ripley 2002), to each of the genotypes in each of the environmental parameters, as implemented in the “splines” *R* package. For this and subsequent analyses, we encoded the different parameter settings as continuous variables; we used splines because some of the strains showed reaction norm shapes that made fitting the same regression model to each of the genotypes inappropriate. The drawback of using splines is that we cannot estimate the critical thresholds when growth rate approaches zero, as natural splines do not allow extrapolation outside of the data range.

### Bayesian estimation of differences between the control and mutant strains

To test for differences between the control and mutant strains in specific environments, we used a Bayesian model analogous to a one-way ANOVA. The model specification followed Gelman (2006) and Kruschke (2011):

$$\mu_{i}=\beta_{0}+\sum_{j}\beta_{j}G_{ji}$$ (3)

where $\beta_{0}$ is the grand mean, $\beta_{j}$ is the effect of the *j*th genotype *G*. For the analysis, we standardized the data such that $\beta_{0}=0$ and $\sum_{j=1}\beta_{j}=0.$ Observations are assumed to be distributed normally around $\mu_{i},$ $y_{i}∼N\left(\mu_{i},\tau_{j}\right),$ where $\tau_{j}$ is a precision of the normal distribution for the *j*th genotype. We used a hierarchical prior for estimating $\beta_{j}$ for each setting of *G* following Gelman (2006), $\beta_{j}∼N\left(0,\tau_{\beta }\right),$ where $\tau_{\beta }=1/\sigma_{\beta }^{2},$ and we used a folded *t*-distribution as a prior for $\sigma_{\beta }.$ Since we are allowing each setting of *G* to have its own variance, $\tau_{j}$ is distributed as $\tau_{j}∼\Gamma \left(s_{G},r_{G}\right),$ where $s_{G}=m^{2}/d^{2},r_{G}=m/d^{2},$ $m∼\Gamma \left(s_{m},r_{m}\right),$ and $d∼\Gamma \left(s_{d},r_{d}\right).$ The benefit of using Bayesian analysis here instead of classical ANOVA is that we allow each genotype to have its own variance, and that there is no special adjustment needed for multiple comparisons as the hierarchical model takes this into account via shrinkage (Gelman 2006; Gelman *et al.* 2012). The model was implemented following Kruschke (2011) with the JAGS program (Plummer 2003), using the R package “rjags” (Plummer 2014). For the MCMC sampling, we used five chains with a burn-in of 10,000 iterations, and sampled 10,000 iterations while thinning by 750 to remove auto-correlation between samples. We checked convergence of the *Markov chain Monte Carlo* (MCMC) simulation through graphical diagnostics.

We calculated contrasts between the control and mutant strains using the posterior distributions for $\beta_{j}$ as $\beta_{\mathrm{mutant}}-\beta_{\mathrm{control}},$ and we also rescaled the $\beta_{j}$ values to their original scale. If a strain is growing slower than the control, its difference will be negative. We considered growth rates of the control and the mutant strains to be significantly different if the 95% highest posterior density (HPD) interval for the difference did not include zero.

### Data availability

Strains are available upon request. File S1 contains all phenotypic data. The authors state that all data necessary for confirming the conclusions presented in the article are represented fully within the article.

## Results

### Growth rates

We found growth of *N. crassa* in our race tubes to be linear: the 95% quantiles for $R^{2}$ values of a linear fit across all measurements were 0.952 – 0.999 with a median of 0.998. The only genotypes exhibiting deviations from a strict linear pattern were *dim-5*, *ngf-1*, and *npf*, with the latter showing the lowest $R^{2}$ in the whole dataset (0.622). This deviation from linear growth was observed particularly in environments where growth was very slow. Because these cases represent only a small portion of the entire dataset, we also used a linear model for the growth of these genotypes. We found that, in the control environment, our control genotype 4200 grew at a rate of 3.29±0.24 (95% CI) mm/hr, in line with previous reports (Ryan *et al.* 1943).

In some tubes where no growth had occurred during the growth assay, we could observe growing mycelium after an extended amount of time (*e.g.*, *npf* in high osmotic stress, and *dim-5* at low temperature). We assigned a growth rate of zero to those measurements.

### Reaction norm experiment

**Figure 1:**
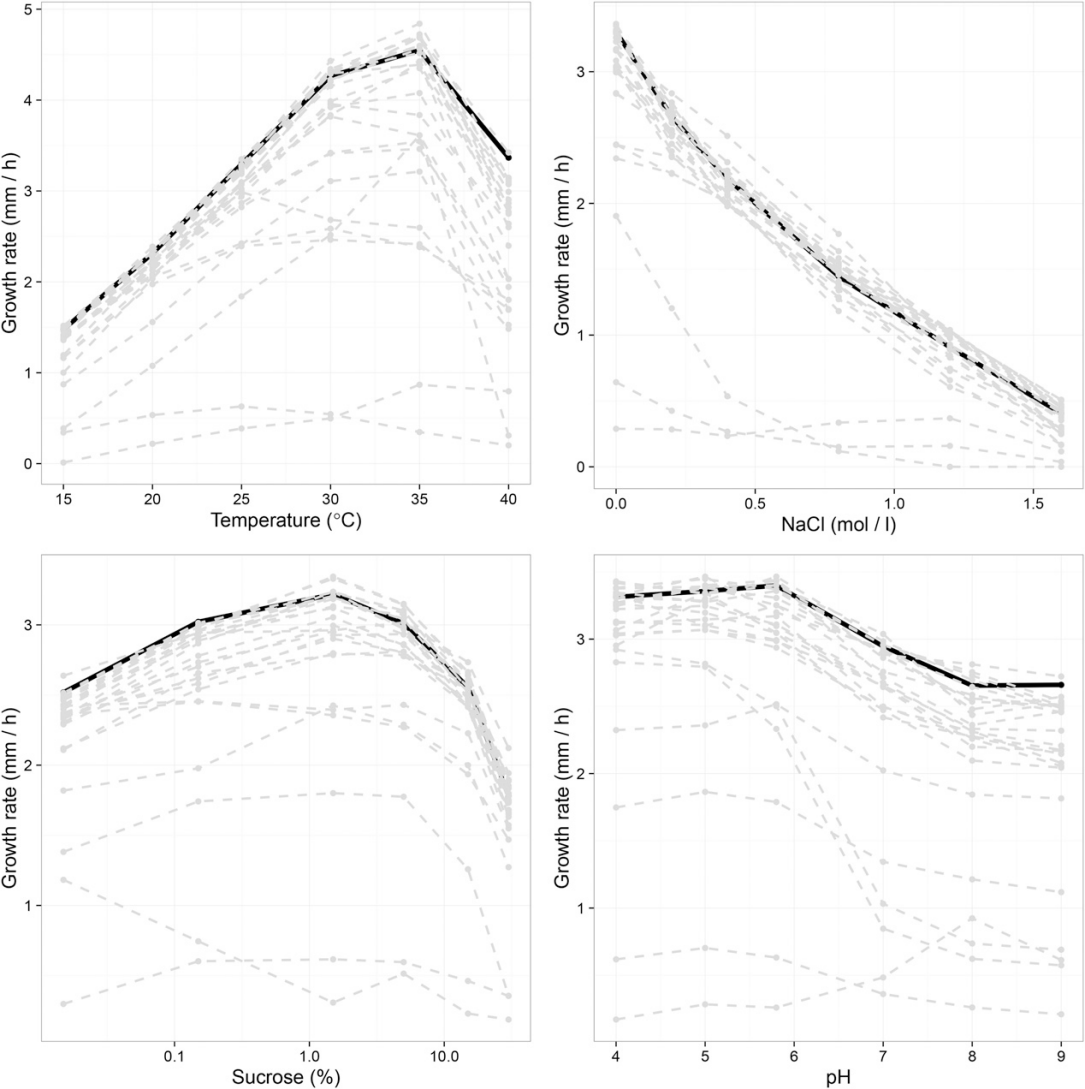
Overview of reaction norms according to each environmental parameter: temperature, osmotic stress, sucrose concentration, and pH (from top left to bottom right). Black reaction norms are the control, and dashed gray lines are the different mutant strains. Nearly all reaction norms are nonlinear. See Figure S1, Figure S2, Figure S3, Figure S4, Figure S5, and Figure S6 for detailed pictures of the different epigenetic mechanisms.

In the reaction norm experiment, data were missing for 19 out of 3120 measurements. Most of these were probably due to failed inoculations, as, in many cases, we were able to distinguish between missing data and no growth in a particular trial. Reaction norms were visualized by plotting growth rate against the different environmental parameters (Figure 1). Visual inspection revealed that nearly all reaction norms were nonlinear, as even in the osmotic stress reaction norms there was some indication of curvature. Even in the osmotic stress environment, the optimum is at zero and growth rate decreases as the salt concentration increases (Figure 1). First, we performed ANOVA to investigate whether the different epigenetic mechanisms have different effects on phenotypic plasticity (Table 2). We did not observe a significant main effect of epigenetic mechanism type, but we did find a significant interaction between epigenetic mechanism and parameter setting nested within stress type, $F_{80,400.17}=1.397,p=0.021$ (Table 2). This result indicates that different epigenetic mechanisms have different effects on different environmental parameters. Thus, subsequently, we analyzed the data by stress type.

**Table 2:** ANOVA of overall differences in reaction norms for all data

	Df, Df2	F Value	P-Value
Fixed effects			
Mechanism	4, 20.02	2.189	0.107
Environmental parameter (E. param.)	3, 60.03	126.668	<2E−16
Environmental setting (within E. param.)	20, 400.37	149.157	<2E−16
Mechanism × Environmental parameter	12, 60.02	1.22	0.291
Mechanism × Environmental setting (within E. param.)	80, 400.17	1.397	0.021
Random effects	$\chi^{2}$	Df	P-value
Genotype	110.6	1	<2E−16
Environmental parameter × Genotype	36.4	1	<2E−9
Environmental setting (within E. param.) × Genotype	1852.1	1	<2E−16

Fixed effects were tested with *F*-tests with Satterthwaite approximation for degrees of freedom, and random effects were tested with $\chi^{2}$-test. For fixed effects: Df, numerator degrees of freedom; Df2, denominator degrees of freedom. There is a significant interaction between epigenetic mechanism and environmental setting within environmental parameter. This indicates that epigenetic mechanisms have different effects in different environmental stresses.

When analyzing the environmental parameters separately, we did not observe a significant main effect of epigenetic mechanism in any of them, and the interaction between mechanism and parameter setting was significant only in the pH trial $F_{20,100.061}=2.271,p=0.004.$ However, the main effect of genotype, and the interaction between genotype and parameter setting, is significant for each parameter (Table 3). These results suggest that epigenetic mechanisms in general contribute to phenotypic plasticity. Among the genotypes, there were differences in both elevation and shape of the reaction norms. We did not see a general effect of particular epigenetic modification type, *e.g.*, all mutant strains with nonfunctional histone methylation have a characteristic reaction norm. On the contrary, we noticed that the reaction norms were specific to a given mutant strain in a particular environment. We also performed the previous analyses excluding genotypes *ngf-1* and *dim-5* as these two genotypes grew much slower in general than the rest, but this did not change any of our conclusions. For the temperature trial, we also investigated whether the two growth chambers used had any different effects on growth. We included growth chamber identity as a fixed factor in the mixed model for the temperature trial, but this term was not significant $F_{1,593.15}=0.545,$ *P* = 0.461, and the growth chamber term was dropped from the final model. We compared the individual mutants to the control with a pairwise ANOVA. We observed that the genotype × environment interaction was significant for many mutants, and these values are reported in Table S3.

**Table 3:** ANOVA of overall differences in reaction norms separately for each environmental parameter

	Temperature			Salt Stress			Sucrose			pH		
Fixed	Df, Df2	F-value	P-value	Df, Df2	F-value	P-value	Df, Df2	F-value	P-value	Df, Df2	F-value	P-value
* M*	4, 20	2.323	0.092	4, 20.009	2.209	0.105	4, 20.007	2.374	0.087	4, 20.005	1.720	0.185
* E*	5, 99.837	130.751	<2E−16	5, 100.203	254.386	<2E−16	5, 100.002	135.925	<2E−16	5, 100.085	65.145	<2E−16
M∗E	20, 99.857	1.107	0.355	20, 100.15	1.629	0.060	20, 100.002	0.755	0.760	20, 100.061	2.271	0.004
Random	$\chi^{2}$	Df	P-value	$\chi^{2}$	Df	P-value	$\chi^{2}$	Df	P-value	$\chi^{2}$	Df	P-value
* G*	106	1	<2E−16	85.7	1	<2E−16	202	1	<2E−16	196	1	<2E−16
G∗E	410	1	<2E−16	1054	1	<2E−16	418	1	<2E−16	319	1	<2E−16

Fixed effects were tested with *F*-tests with Satterthwaite approximation for degrees of freedom and random effects were tested with $\chi^{2}$-test. For fixed effects: Df, numerator degrees of freedom; Df2, denominator degrees of freedom. The main effect of genotype and the interaction between genotype and environmental setting is significant for each environmental stress type. This suggests that in general epigenetic mechanisms contribute to phenotypic plasticity. *M*, Mechanism; *E*, Environmental setting; *G*, Genotype.

**Figure 2:**
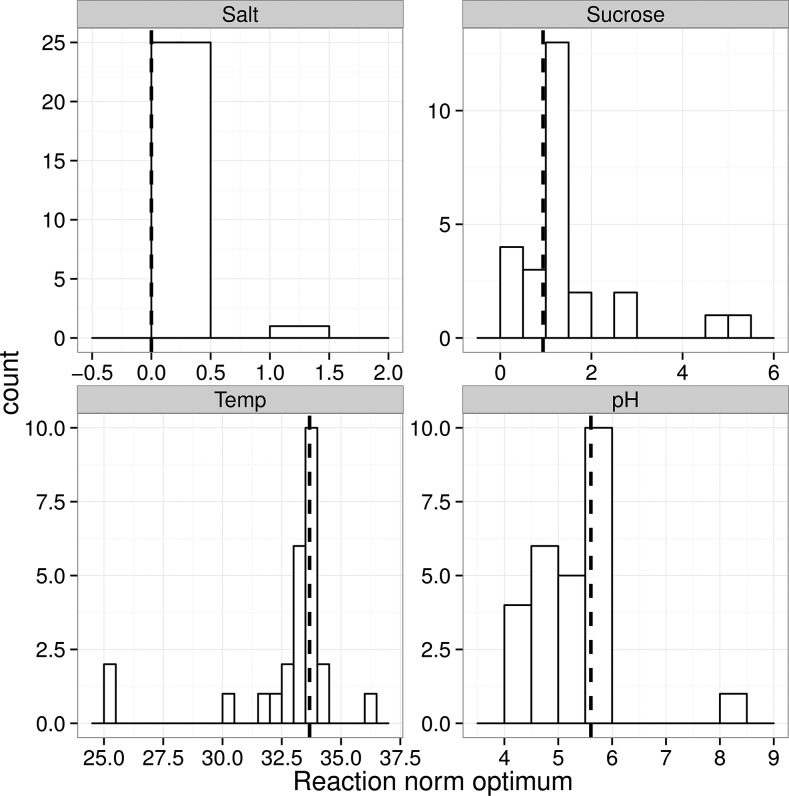
Distributions of the optimal settings for the mutant strains for the four environmental parameters. An optimal environment was estimated from a natural spline fit to the reaction norm data for each strain. Dashed vertical lines indicate the optimal parameter setting for the control. Changes in reaction norm optima happened in all environments expect salt stress, where the one observation with a different optimum is a mutant that grew very poorly, and had a nearly flat reaction norm.

We also observed that there were changes in reaction norm optima among the mutant strains, as calculated from the natural spline fits. Plotting the distribution for the optimal environment of each genotype shows that osmotic stress is the only parameter where the optimum remains constant at 0 M NaCl for all genotypes (Figure 2), except for *dim-5*, where the reaction norm is rather flat (Figure S2). In the other environments, some genotypes have a different environmental optimum than the control (Figure 2).

**Figure 3:**
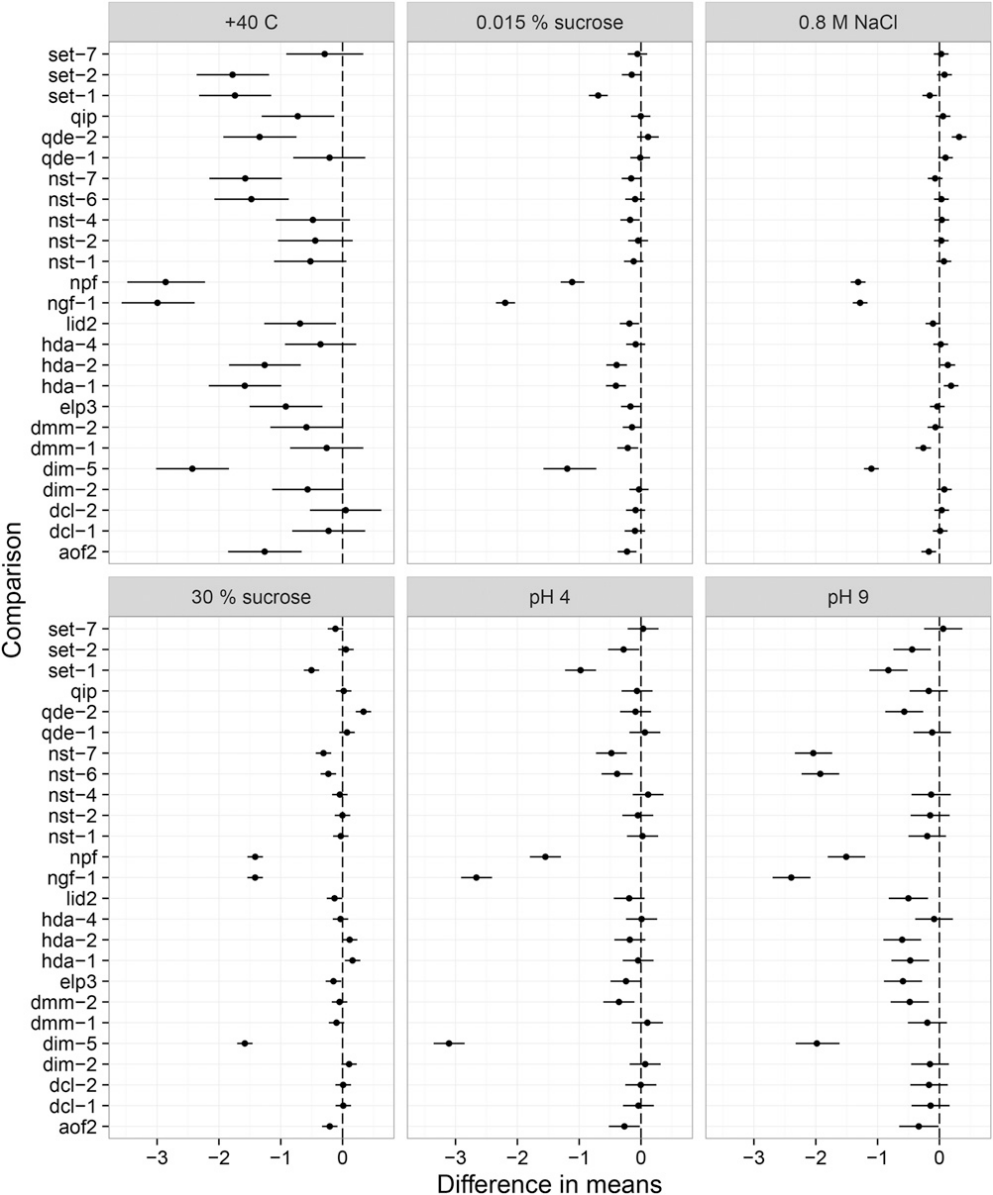
Contrasts for the mutant growth rates in different environments. These particular environmental settings were chosen as examples to the figure, as they contain many cases where the mutants differ from the control. Vertical axis shows the different mutants, and the horizontal axis shows the mutant minus control difference in mean growth rates; error bars, 95% HPD intervals. Vertical lines in the panels show the difference of zero line. Many mutants are significantly different from the control, in the 40° environment variation is greater than in the other environments.

Having established that there are statistically significant differences among the mutant strains in the different environments, we now present the results according to each epigenetic mechanism, and by parameter, highlighting interesting mutants and their effects. Reaction norms for all the different classes separately can be found in Figure S1, Figure S2, Figure S3, Figure S4, Figure S5, and Figure S6. Figure 3 shows differences of the mutants to the control, for simplicity, in selected environmental settings. These environmental settings were selected because they contain many cases where mutants differ from the control. A summary of the results for each mutant can be found in Table 4.

**Table 4:** Summary of the results synthesizing the results of the reaction norm experiment and the validation experiments

Gene	Temperature	Salt Stress	Sucrose Concentration	pH
*dim-2*	No effect	No effect	Minor shape change at 30% sucrose	No effect
*dmm-1*	No effect	Lower elevation	Lower elevation	Lower elevation
*dmm-2*	Lower elevation	Lower elevation	Lower elevation, minor shape change	Lower elevation, minor shape change
*dim-5*	Poor growth	Poor growth	Poor growth	Poor growth
*set-1*	Lower elevation	Lower elevation	Lower elevation, minor shape change	Shape change, lower elevation
*set-2*	Shape change	No effect	Minor shape change	Lower elevation
*set-7*	No effect	No effect	No effect	No effect
*npf*	Lower elevation, shape change	Lower elevation, poor growth	Lower elevation	Lower elevation
*nst-1*	No effect	No effect	Lower elevation	No effect
*nst-2*	No effect	No effect	No effect	No effect
*nst-4*	No effect	No effect	No effect	No effect
*nst-6*	Lower elevation, shape change	Lower elevation	Lower elevation, shape change	Lower elevation, shape change
*nst-7*	Lower elevation, shape change	Lower elevation	Lower elevation, shape change	Lower elevation, shape change
*hda-1*	Lower elevation, minor shape change	Change in elevation	Lower elevation, minor shape change	Lower elevation
*hda-2*	Lower elevation, minor shape change	Change in elevation	Lower elevation, minor shape change	Lower elevation
*hda-4*	No effect	No effect	No effect	No effect
*qde-1*	No effect	No effect	Minor elevation change	No effect
*qde-2*	Lower elevation, shape change	Elevation change	Shape change	Lower elevation, minor shape change
*dcl-1*	No effect	No effect	No effect	No effect
*dcl-2*	No effect	No effect	No effect	No effect
*qip*	No effect	No effect	No effect	No effect
*aof2*	Lower elevation	Lower elevation	Lower elevation	Lower elevation
*elp3*	Lower elevation	Lower elevation	Lower elevation	Lower elevation
*lid2*	Lower elevation	Lower elevation	Lower elevation	Lower elevation
*ngf-1*	Poor growth	Poor growth	Poor growth	Poor growth

Descriptions in the table are changes of the reaction norms of the mutant strains relative to the control.

### Effects of DNA methylation

So far, no apparent phenotype other than the lack of methylation has been detected for the DNA methyltransferase mutant *dim-2*. In our experiment, *dim-2* failed to demonstrate any observable phenotypic effects in the pH, salt, or sucrose trials. In the temperature trial, *dim-2* had no effect up to 35°, but grew slower than the control at 40°, with a difference of −0.57 (−1.14 to −0.02, 95% HPD) mm/hr, and we sought to validate this suggestive difference. In the validation experiment, we observed that *dim-2* grew at the same rate as the control with a difference of 0.01 (−0.17 to 0.19, 95% HPD) mm/hr. Thus, we conclude that lacking a DNA methylation system has no effect at 40°. In the 30% sucrose setting, we first observed a nonsignificant tendency of *dim-2* to grow faster than the control (Figure S1), and we confirmed this finding in a validation experiment, where we observed a small but significant effect; with a difference of 0.07 (0.03 to 0.12) mm/hr.

The *dmm-1* and *dmm-2* mutants control the propagation of DNA methylation from heterochromatic regions (Honda *et al.* 2010). We observed that while *dmm-1* had the same optimum sucrose concentration as the control, it grew slower at lower sucrose concentrations (Figure S1); with a difference of −0.21 (−0.38 to −0.05) mm/hr at 0.015% sucrose—a reduction of 8%. It also grew slower at higher pH, and the reaction norm had a lower elevation in the salt stress trial. Generally, *dmm-2* had a slightly lower elevation for all reaction norms (Figure S1). In the pH trial, its reaction norm had a different shape compared to the control, and the optimal setting of *dmm-2* was 5.0 compared to 5.8 of the control, and the difference in growth rates at pH 4 was −0.36 (−0.61 to −0.11) mm/hr. We validated the growth of *dmm-2* in pH 4 and pH 9; at pH 4 the difference in growth was −0.43 (−0.50 to −0.36) mm/hr, and at pH 9 the difference was −0.61 (−0.70 to −0.51) mm/hr, confirming the phenotypic response to different pH for *dmm-2*. Thus, the *dmm* mutant strains had different phenotypic responses. The results suggest that (possibly silenced) genes adjacent to heterochromatic regions control the response to several environmental parameters.

Overall, our data suggest that DNA methylation does not play a very important role in phenotypic plasticity, but spurious DNA methylation has the potential to affect phenotypic plasticity.

### Effects of histone methylation

Histone methylation has multiple functions depending on which residues are methylated (Rothbart and Strahl 2014). For the *set-7* mutant, which lacks H3K27me3, we did not observe any growth responses among the environmental parameters and settings we tested (Figure S2). The *npf* mutant also lacks H3K27me3, but is also impaired in other functions (Jamieson *et al.* 2013). Generally, *npf* grew much slower than the control; in the sucrose and pH trials, the differences were seen in reaction norm elevation rather than shape (Figure S2), but, in the temperature and salt stress trials, *npf* presents a different shape. In particular, *npf* seems to be sensitive to high temperatures and osmotic stress, as its growth rate collapses at 40° and in high salt (Figure S2). However, these changes are not related to H3K27me3 as *set-7* does not show them.

The *set-1* mutant lacks H3K4me3, and shows a lower elevation of its reaction norms and also shape changes (Figure S2). In the sucrose and pH trials, growth of *set-1* slows down more than the control when the pH is changed from 5.8 to pH 4.0, a difference of 0.19 (−0.05 to 0.43) mm/hr between pH 5.8 and pH 4.0, but for the control the difference is only 0.09 (−0.15 to 0.32) mm/hr, although this is only marginally significant. This can also be observed when going from 1.5 to 0.15% sucrose; for *set-1* the difference is 0.37 (0.17 to 0.58) mm/hr, while for the control the difference is only −0.08 (−0.28 to 0.12) mm/hr. As H3K4me3 has been implicated in transcriptional activation (Pokholok *et al.* 2005; Raduwan *et al.* 2013), some genes may not activate correctly in these environments.

H3K36me is believed to be required for efficient transcriptional elongation (Morris *et al.* 2005). For the *set-2* mutant, which lacks H3K36me, we observed a generally lower elevation for reaction norms (Figure S2), but not to the same extent as *set-1*. However, *set-2* has a markedly different response to temperature, as its optimal setting from a natural spline fit is at 25.3° Compared to the 33.7° of the control (Figure S2). This suggests that H3K36me is involved in the transcription of genes required for a high temperature response.

The final histone modification we investigated was H3K9me; the mutant *dim-5* that lacks this modification (Tamaru and Selker 2001; Tamaru *et al.* 2003) grew very poorly in all environments (Figure S2), as noted previously (Tamaru and Selker 2001). Thus, H3K9me plays a central role in essential cellular processes.

Taken together, our results show that H3K27 trimethylation has no observable phenotypic effect, H3K4 trimethylation and H3K36 methylation have some effects on elevation of reaction norms but also on their shapes, while H3K9 methylation is needed for normal cellular function.

### Effects of histone deacetylation

For histone deacetylation, we used two different classes of mutants: *hda-1*, *hda-2*, and *hda-4*, and type III (NAD^+^ dependent) histone deacetylases *nst-1*, *nst-2*, *nst-4*, *nst-6*, and *nst-7*. We observed that, for the *hda* mutants, reaction norm elevations were reduced (Figure S3), except in the salt stress trial, where *hda-1* and *hda-2* grew faster than the control (Figure 3). The difference in growth between *hda-1* and the control in 0.8 M NaCl was −0.19 (0.07 to 0.31), an increase of 13%. Such an increase could also be observed in the 30% sucrose environment.

For the *nst* mutants 1, 2, and 4, we did not observe any notable phenotypic effects. The two remaining *nst* mutants (6 and 7) showed large growth effects in nearly all trials. Their effects were particularly noticeable in the pH trial, where growth was drastically reduced at high pH (Figure 3 and Figure S4), and both of their optimal environments were at lower pH settings than for the control: pH 4.0 for *nst-6* and pH 4.5 for *nst-7* compared to the optimum of pH 5.6 for the control. Also, their reaction norms to sucrose at low concentrations were flat; they did not increase their growth rate in response to rising sucrose concentrations from 0.015% sucrose as the control does.

Thus, *hda-1* and *hda-2* generally presented a lower reaction norm elevation similar to *nst-6* and *nst-7*, which also had a different reaction norm shape. Otherwise, histone deacetylation mutants showed no observable responses in terms of their reaction norms and the parameters and settings we tested.

### Effects of RNA interference

For the RNA interference mutants we did not observe any effects of the two Dicer ribonuclease genes, *dcl-1* and *dcl-2*, that are involved in micro RNA maturation, or the RNA-polymerase encoding *qde-1* gene, associated with initiation of the RNA interference pathway (Figure S5). For the *qip* mutant, which encodes an exonuclease involved in siRNA maturation, we saw a phenotype effect only in the 40° environment, where the difference from the control was −0.72 (−1.31 to −0.13) mm/hr. However, when we attempted to replicate this finding in a validation experiment, we did not observe any significant differences between *qip* and the control; the difference was 0.03 (−0.15 to 0.21) mm/hr. Thus, we conclude that *qip* has no effect at 40°.

However, we observed a different reaction norm shape in all trials with *qde-2*, a gene encoding the *Neurospora* Argonaute protein, which is an essential component of micro RNA processing (Figure S5). Growth was faster than the control at intermediate salt concentrations (Figure 1); for instance, in 0.8 M NaCl, the difference in growth rate to the control was 0.32 (0.20 to 0.44) mm/hr—an increase of 22%. An increase in growth rate compared to the control was also observed in the 30% sucrose environment, a difference of 0.34 (0.21 to 0.46) mm/hr. We confirmed this result in a validation experiment where we observed a difference of 0.33 (0.28 to 0.38) mm/hr. The growth rate of *qde-2* also had a tendency to increase in the 0.015% sucrose environment, and although this effect was not significant in our first experiment, we observed a significant increase in the validation experiment; a difference of 0.10 (0.05 to 0.16) mm/hr. Based on natural spline fit, the *qde-2* mutant strain also had a lower optimal temperature of 31.9° (*vs.* 33.7°), and we validated this result by measuring backcrossed *qde-2* at 30 and 35°. We observed that the shape of the *qde-2* reaction norm did indeed change, and the growth rate of *qde-2* increased by 0.29 (0.18 to 0.41) mm/hr compared to an increase of 0.68 (0.57 to 0.79) mm/hr when the temperature increased from 30 to 35°. This indicates that small RNA molecules that are QDE-2-dependent (Lee *et al.* 2010) are involved in the response to high temperatures. For the pH trial, elevation of the *qde-2* reaction norm was generally lower, but, at pH 9, a *qde-2* shape change was indicated (Figure S5). We validated the growth difference between the control and *qde-2* at pH 9, and found a difference of −0.59 (−0.69 to −0.49) mm/hr.

In summary, for the RNA interference pathway, only *qde-2* showed a different reaction norm; other mutant strains did not show any effects.

### Effects of histone demethylation and acetylation

The viable histone acetyltransferase mutant *ngf-1* grew poorly (Figure S6), indicating the critical role played by this gene in normal cellular function. The other mutant, *elp3*, presented reaction norms with slightly lower elevations in all trials. We observed that the *elp3* mutant grew slower at high temperatures. We also observed that its pH optimum dropped from 5.6 to 4.9.

The two putative histone demethylases (*lid2* and *aof2*) had lower elevation in their reaction norms for all environments (Figure S6). However, the reaction norm shape of *lid2* changed as the temperature decreased to an optimum of 32.0°, but this result could not be validated as there was no significant difference in the change in growth rate between control and *lid2* when the temperature increased from 30 to 35°. In contrast, *aof2* had a growth rate that was indistinguishable from the control at 35°, but its growth rate dropped dramatically when the temperature increased to 40° (Figure 3 and Figure S6). The difference in growth rate between the control and *aof2* in 40° was −1.26 (−1.85 to −0.66) mm/hr, a drop of 38%. We validated this result and observed that *aof2* grew slower than the control in the validation experiment at 40° as well; with a difference of −0.38 (−0.57 to −0.18) mm/hr. However, this growth response was less obvious in the validation experiment, where the change in growth rate was not significantly different from the control. Therefore, we conclude that *aof2* and *lid2* are not important to the temperature-dependent responses of *N. crassa*.

In conclusion, the effects of histone acetylation on reaction norms were mainly on reaction norm elevation, and presented only slight changes to shape, while histone demethylation affected mainly the elevation of reaction norms.

## Discussion

While epigenetic mechanisms have been shown to be important in certain plastic responses (Slepecky and Starmer 2009; Bossdorf *et al.* 2010; Herrera *et al.* 2012; Baerwald *et al.* 2016), the extent to which they contribute to phenotypic plasticity, or how they maintain homeostasis in organisms facing changing environments has been largely unexplored. By exposing a set of deletion mutants of the filamentous fungus *N. crassa* to a spectrum of controlled environmental parameters, we showed that certain epigenetic modifications have strong effects on plasticity, while others do not. In our experiment, epigenetic modifications affected the sensitivity to environmental change, and, to a lesser extent, growth of the mutant strain. There remains the theoretical possibility that some phenotypic changes could be due to changes in genetic background, as we did not test backcrossed strains of all mutants, but this possibility is remote as the genetic background of the control and the mutants is nearly isogenic as described in *Materials and Methods*. Modification types did not have a consistent pattern in their effects on phenotype. However, it may be that our classification of epigenetic mechanism was too coarse, and this may be why we did not observe a consistent effect. For instance, histone methylation at different residues are known to have different effects (Greer and Shi 2012), and it is also possible that there is redundancy between different epigenetic mechanisms. Thus, it may be that no general effects exists for a certain type of modification as a group. Instead, phenotypic effects were specific to the epigenetic modification in a given environment.

Epigenetic mechanisms clearly played a role in the phenotypic plasticity of growth according to several environmental variables, corroborating recent suggestions concerning the epigenetic control of phenotypic plasticity (Schlichting and Wund 2014). One of the main findings of this study was that epigenetic modifications were more important for plasticity than average growth rate, *i.e.*, genotypes differed less in terms of their average growth than their variances across the environments. This indicates that plasticities in different environments are caused by different epigenetic mechanisms. Thus, we find no evidence of loci that universally affect plasticity, but instead found that plasticity, and thus gene expression, are context dependent Windig *et al.* (2004), as different epigenetic mechanisms affect plasticity in different environments.

### Histone modifications

Our results suggest that histone modifications play an important role in how *N. crassa* responds to environmental perturbation. Histone modifications H3K36me and H3K4me3 are important in plastic responses to temperature and pH, respectively. *Set-2* is responsible for H3K36 methylation, and the strain lacking a functional form of this gene suffered some developmental deficiencies, *i.e.*, female sterility, and production of few conidia (Adhvaryu *et al.* 2005). It also grew more slowly than the wild type in most environments, but especially so at high temperatures. The optimum growth rate of *set-2* is at 25°, while the wild type has an optimum at 35°. This indicates that H3K36 methylation is required for the correct expression of genes required at temperatures above 25°. In other organisms, H3K36 methylation has been associated with transcriptional elongation (Morris *et al.* 2005; Hampsey and Reinberg 2003); H3K36me is present in the active regions of eukaryotic genomes, and its function seems to be to keep the chromatin of actively transcribed genes open (Venkatesh *et al.* 2012). It may be that genes expressed under certain environmental circumstances need to be kept in open conformation by H3K36me, and the nonfunctional strain clearly had a problem at high temperatures. The gene responsible for H3K4 trimethylation, *set-1*, is important for the response to pH. Previously, it has been reported that H3K4me3 is needed for correct expression of the circadian clock gene *frq* in *N. crassa* (Raduwan *et al.* 2013). In general, H3K4me3 is associated with the 5′-regions of actively transcribed genes (Pokholok *et al.* 2005; Ardehali *et al.* 2011). In *N. crassa*, *set-1* has a growth phenotype, suggesting that H3K4me3 is needed for normal cellular metabolism as well as a specific response to acidic pH. In contrast to *set-1* and *set-2*, the *set-7* mutant strain did not present any phenotypic effect. The gene *set-7* is responsible for H3K27 trimethylation, and genes marked with H3K27me3 are silent in *N. crassa* (Jamieson *et al.* 2013). Genes marked with H3K27me3 tend to be less conserved, suggesting that they are needed only in certain environmental conditions. Therefore, it is surprising to observe that the *set-7* mutant strain performed as well as the wild type in our trials. It may be that a lack of repression by H3K27me3 (which allows genes to be expressed) does not prevent a plastic response. The *npf* mutant also lacks H3K27me3 (Jamieson *et al.* 2013); however, it has a very different phenotype from that of *set-7*, and severe growth defects, which cannot be attributed to lack of H3K27me3. Furthermore, H3K9 methylation seems essential for normal cellular function, as H3K9me lacking *dim-5* mutant (Tamaru and Selker 2001; Tamaru *et al.* 2003) had a severe growth defect in all trials. This phenotype is possibly due to the role of H3K9me in genome integrity (Lewis *et al.* 2010a).

### DNA methylation

DNA methylation in *Neurospora* is directed at regions where histone 3 lysine 9 methylation (H3K9me) is present. H3K9me is required for DNA methylation as *dim-5* lacks both H3K9me and the ability to perform DNA methylation (Tamaru and Selker 2001; Tamaru *et al.* 2003). DNA methylation can cause gene silencing in *Neurospora* as growth defects of *dmm* mutants were alleviated after the removal of DNA methylation (Honda *et al.* 2010), and DNA methylation can also cause silencing of antibiotic resistance genes (Lewis *et al.* 2010b) but is not required for all gene silencing (Honda *et al.* 2012). However, a complete lack of DNA methylation in the *dim-2* mutant was associated with only a slight response in the 30% sucrose environment. This is in stark contrast to land plants and vertebrates, where DNA methylation appears to be indispensable (Li *et al.* 1992; Rai *et al.* 2006; Xiao *et al.* 2006; Yaari *et al.* 2015). We observed lower elevation of the reaction norms for *dmm-1* and *dmm-2* mutants in all environments, and a change in reaction norm shape for *dmm-2* in response to pH (*i.e.*, poor growth at pH 4), suggesting that genes required for this response are silenced as DNA methylation spreads from heterochromatic regions in these mutant strains (Honda *et al.* 2010).

### Histone deacetylation

We examined two different classes of histone deacetylase genes: the Class I histone deacetylases, and NAD^+^ dependent Class III histone deacetylases. Class I includes the *hda* genes: *hda-1*, *hda-2*, and *hda-4*. Although it has been reported that histone H2B is the main target of HDA-1, it can also deacetylate H3, and is involved in controlling DNA methylation (Smith *et al.* 2010; Honda *et al.* 2012). HDA-4 broadly increases acetylation of histones H3 and H4, and no marked effects were reported for the *hda-2* mutant (Smith *et al.* 2010). Therefore, it is striking that the phenotypes of *hda-1* and *hda-2* are very similar. These knockout strains presented reaction norms with a similar shape to the wild type in all environmental trials other than osmotic stress (where they grew faster), but with a lower elevation, indicating that normal cellular functioning is impaired. Enhanced growth in osmotic stress is surprising, and one interpretation is that there is some cost associated with expressing these genes in this environment. On the other hand, *hda-4* was no different from the control, indicating that it is not involved in phenotypic plasticity in the environments tested here. Class III histone deacetylases include the *nst* genes: *nst-1*, *nst-2*, *nst-4*, *nst-6*, and *nst-7*; these are homologous to the SIR2 family of histone deacetylases (Blander and Guarante 2004). In *N. crassa*, it has been shown that *nst-1* and *nst-2* are involved in telomeric silencing (4, 6, and 7 were not examined), and that NST-1 deacetylates H4K16 (Smith and Kruglyak 2008). In other eukaryotes, sirtuin proteins can have targets other than histones (Blander and Guarante 2004), so, for *nst-4*, *nst-6*, and *nst-7*, we cannot be certain of their functions. In trials, *nst-1*, *nst-2*, and *nst-4* did not present any growth effect in the environments tested. However, the reaction norms of *nst-6* and *nst-7* had pronounced shape changes in sucrose concentration and pH trials. Moreover, the reaction norms of these two mutants were very similar, suggesting that they may work in a similar way.

### RNA interference pathway

RNA interference in *Neurospora* may not be strictly an epigenetic mechanism. The canonical RNA interference pathway is not required for DNA methylation (Freitag *et al.* 2004), and it is not known if RNA-directed epigenetic modifications, such as the plant RNA-directed methylation pathway (Matzke and Mosher 2014), exist or if RNA molecules can mediate epigenetic inheritance like in animals (Rassoulzadegan *et al.* 2006; Rechavi *et al.* 2011; Ashe *et al.* 2012) or other fungi (Qutob *et al.* 2013; Calo *et al.* 2014). However, a class of small RNAs called disiRNAs, may be involved in controlling DNA methylation at specific loci (Dang *et al.* 2013). Therefore, the possibility of RNA-mediated epigenetic effects exists, so we included appropriate genes in our examination of the system. We observed that the *N. crassa* ARGONAUTE (Meister 2013) homolog QDE-2 was involved in multiple responses to environmental stress, while the two Dicer protein homologs (DCL-1 and DCL-2), QDE-1, and QIP were not. In *N. crassa*, there are several pathways that generate different kinds of small RNAs: the biogenesis of siRNAs is dependent on Dicer proteins, QDE-2, and QIP (Maiti *et al.* 2007); qiRNAs are Dicer- and QDE-1-dependent, and are involved in the DNA damage response (Lee *et al.* 2009); disiRNAs are Dicer-independent small interfering RNAs that are generated from loci that have overlapping sense and antisense transcripts (Lee *et al.* 2010; Dang *et al.* 2013); and microRNA-like RNAs (milRNAs) that can silence genes and are generated by multiple different mechanisms (Lee *et al.* 2010). The biogenesis of some milRNAs requires QDE-2 (Lee *et al.* 2010), other milRNAs are Dicer-dependent and QDE-2-independent, and some require QIP while others do not (Lee *et al.* 2010). While it remains possible that some Dicer-dependent small RNAs are produced by *dcl-1* and *dcl-2*, as these genes are, at least partially, redundant (Catalanotto *et al.* 2004), our results suggest that those small RNAs that are QDE-2-dependent are involved primarily in plastic responses to the environment.

### Histone demethylation and acetylation

Of the remaining genes, *ngf-1* and *elp3* are believed to encode histone acetyl transferases based on their similarity to those genes in yeast (Wittschieben *et al.* 1999; Brenna *et al.* 2012). In *N. crassa*, Brenna *et al.* (2012) showed that NGF-1 is involved in transducing environmental signals, but we found that the *ngf-1* mutant grew very slowly in all environments, indicating that key cellular processes are impaired. The *elp3* mutant grows slower and its reaction norms have generally lower elevation, but there was no indication of a shape change. Previously, it has been reported that yeast *elp3* has a temperature-sensitive phenotype (Wittschieben *et al.* 1999). Indeed, we observed that differences in growth between *elp3* and the wild type were largest at 40°. However, *elp3* still has the same temperature optimum (35°) as the wild type, suggesting that rather than a temperature response itself, a more fundamental biological process is impaired in the *elp3* mutant strain. The genes *aof2* and *lid2* are inferred to encode histone demethylases. In fission yeast, the *N. crassa* AOF2 protein homolog LSD1 acts as a histone demethylase that demethylates H3K4me and H3K9me (Lan *et al.* 2007). We observed *aof2* reaction norms with lower elevation in salt stress, sucrose concentration, and pH trials. This suggests that certain cellular processes are not functioning normally. The yeast homolog of LID2 also acts as a H3K4 demethylase, and interacts with the H3K9 methylation complex (Li *et al.* 2008). The phenotype of the *lid2* mutant is similar to *aof2* in that it had lowered reaction norm elevation in all trials, although its reaction norm shape appears similar to wild type.

### Conclusions

In terms of the different environmental parameters tested, we observed that epigenetic mechanisms in *N. crassa* play a much greater role in the response to temperature and pH changes than they do in the response to shifts in sucrose concentration and osmotic stress. This can be explained by the ecology of *Neurospora*, and considering that it is a saprotrophic fungus found in dead plant matter (Jacobson *et al.* 2006), or can act as an endophyte under certain conditions (Kuo *et al.* 2014). Temperature changes are the most common environmental variable that organisms experience, and pH changes are likely to occur as the fungus encounters different substrates in nature. It may be that *N. crassa* rarely encounters elevated NaCl levels in a terrestrial environment, and has not evolved a plastic response to it. In the sucrose concentration trial, we examined how the level of available nutrients and osmotic stress affect the growth of *N. crassa*, and it would be interesting to investigate how the fungus responds to different types of carbon sources, and whether those responses are under epigenetic control.

Another question that requires investigation is whether the plastic responses we have detected are heritable. It has been observed that maternal or transgenerational effects can be mediated mechanistically by epigenetic changes. In plants, DNA methylation and RNA-directed methylation, in particular, have been implicated in transgenerational inheritance (Luna *et al.* 2012; Luna and Ton 2012). In fruit flies, histone modifications have also been linked to transgenerational inheritance, where H3K27 and H3K9 methylation regulate offspring lipid content in response to paternal diet (Öst *et al.* 2014).

Our results show that epigenetic mechanisms are involved in plastic responses of *N. crassa*, and that histone methylation is likely to be the main mechanism, along with small RNAs that are dependent on QDE-2. We suggest that epigenetic mechanisms are likely to be important mediators of plastic responses. Epigenetic mechanisms may also facilitate evolutionary adaptation via phenotypic plasticity, as suggested by models (Lande 2009; Chevin *et al.* 2010; Draghi and Whitlock 2012) and experiments (Schaum and Collins 2014; Lind *et al.* 2015). The role of epigenetic mechanism remains unknown until we have determined whether, and how often, plasticity occurs across generations.

## Supplementary Material

Supplemental Material
