# Supplementary material for: Epigenetic Control of Phenotypic Plasticity in the Filamentous Fungus Neurospora crassa
Source: G3 (Bethesda). 2016 Sep 29;6(12):4009–22. doi: 10.1534/g3.116.033860 (PMC5144970; doi:10.1534/g3.116.033860)
Supplement: Supplemental Material [file supp_6_12_4009__index.html]

Epigenetic Control of Phenotypic Plasticity in the Filamentous Fungus Neurospora crassa — Supplemental Material 

# Epigenetic Control of Phenotypic Plasticity in the Filamentous Fungus *Neurospora crassa*

## Supplemental Material for Kronholm, *et al*, 2016

**Files in this Data Supplement:**

- Table S1 - *Neurospora* strains used in this study. (.pdf, 64 KB)
- Table S2 - PCR primers used in this study. (.pdf, 51 KB)
- Table S3 - Results of pairwise ANOVA comparing all mutants to the control in the different environments. (.pdf, 56 KB)
- Figure S1 - Reaction norms for DNA methylation mutants in the four environmental parameters. (.eps, 53 KB)
- Figure S2 - Reaction norms for histone methylation mutants in the four different environmental parameters. (.eps, 70 KB)
- Figure S3 - Reaction norms for histone deacetylation mutants in the four environmental parameters. (.eps, 52 KB)
- Figure S4 - Reaction norms for histone deacetylation, type III mutants in the four different environmental parameters. (.eps, 70 KB)
- Figure S5 - Reaction norms for RNA interference mutants in the four different environmental parameters. (.eps, 71 KB)
- Figure S6 - Reaction norms for histone acetylation and putative histone demethylase mutants in the four different environmental parameters. (.eps, 62 KB)
- File S1 - Phenotypic data. (.xlsx, 812 KB)
