# Supplementary material for: Epigenetic Control of Phenotypic Plasticity in the Filamentous Fungus Neurospora crassa
Source: G3 (Bethesda). 2016 Sep 29;6(12):4009–22. doi: 10.1534/g3.116.033860 (PMC5144970; doi:10.1534/g3.116.033860)
Supplement: Supplemental Material [file supp_g3.116.033860_TableS1.pdf]

Table S1: *Neurospora* strains used in this study. Strains used in the reaction norm experiment are indicated as are the strains used in the validation experiments. Strains marked as preparation were used for generating some of the strains used in the experiments. HET = Heterokaryon with wild type.

Genotype	Mating type	Source	Strain ID	Experiment
Δ*dim-2::hph*	*mat a*	Selker lab	N1850	reaction norms
Δ*dmm-1::hph*	*mat a*	FGSC	14504	reaction norms
Δ*dmm-2::hph*	*mat a*	FGSC	11100	reaction norms
Δ*dim-5::hph*	*mat a*	this study	K1	reaction norms
Δ*set-1::hph*	*mat a*	this study	K2	reaction norms
Δ*set-2::hph*	*mat a*	FGSC	15504	reaction norms
Δ*set-7::hph*	*mat a*	FGSC	11182	reaction norms
Δ*npf::hph*	*mat a*	FGSC	13915	reaction norms
Δ*nst-1::hph*	*mat a*	FGSC	12403	reaction norms
Δ*nst-2::hph*	*mat a*	FGSC	12078	reaction norms
Δ*nst-4::hph*	*mat a*	FGSC	11165	reaction norms
Δ*nst-6::hph*	*mat a*	FGSC	22669	reaction norms
Δ*nst-7::hph*	*mat a*	FGSC	16002	reaction norms
Δ*hda-1::hph*	*mat a*	FGSC	12003	reaction norms
Δ*hda-2::hph*	*mat a*	FGSC	11158	reaction norms
Δ*hda-4::hph*	*mat a*	FGSC	11175	reaction norms
Δ*qde-1::hph*	*mat a*	FGSC	11156	reaction norms
Δ*qde-2*	*mat a*	Selker lab	N2271	reaction norms
Δ*dcl-1::hph*	*mat a*	FGSC	15892	reaction norms
Δ*dcl-2::hph*	*mat a*	FGSC	11155	reaction norms
Δ*qip::hph*	*mat a*	FGSC	12130	reaction norms
Δ*aof2::hph*	*mat a*	FGSC	11964	reaction norms
Δ*elp3::hph*	*mat a*	FGSC	11976	reaction norms
Δ*lid2::hph*	*mat a*	FGSC	11876	reaction norms
Δ*ngf-1::hph*	*mat a*	FGSC	16229	reaction norms
wt	*mat a*	FGSC	4200	reaction norms
wt	*mat A*	FGSC	2489	validation
Δ*dim-2::hph* BC_5 2489	*mat A*	this study	K3	validation
Δ*dmm-2::hph* BC_5 2489	*mat A*	this study	K4	validation
Δ*hda-1::hph* BC_5 2489	*mat A*	this study	K5	validation
Δ*qde-2* BC_5 2489	*mat A*	this study	K7	validation
Δ*qip::hph* BC_5 2489	*mat A*	this study	K8	validation
Δ*aof2::hph* BC_5 2489	*mat A*	this study	K9	validation
Δ*lid2::hph* BC_5 2489	*mat A*	this study	K10	validation
Δ*set-7::hph* BC_6 2489	*mat A*	this study	K11	validation
Δ*set-1::hph*	*mat A*	FGSC	15828	preparation
Δ*dim-5::hph* HET	*mat a*	FGSC	15885	preparation
