# Supplementary material for: Epigenetic Control of Phenotypic Plasticity in the Filamentous Fungus Neurospora crassa
Source: G3 (Bethesda). 2016 Sep 29;6(12):4009–22. doi: 10.1534/g3.116.033860 (PMC5144970; doi:10.1534/g3.116.033860)
Supplement: Supplemental Material [file supp_g3.116.033860_TableS2.pdf]

Table S2: PCR primers used in this study.

Locus	Forward	Reverse	Annealing temp. (°C)
dim-2	TTGGAAAGGTGTTGCGTCTG	GTGATCATCCGTGTCCGTTG	67
dmm-1	TGGATGACGCTTTGCAACAA	CGGATCAGGATAAGCGGACT	66
dmm-2	GGTTACGGCAGTTTCGGATC	TAATAGCCTCGCCCCTGATG	66
dim-5	TATTCACGACCTTGCCCTGT	CAATGATTCGGGCGCAGTAA	65
set-1	TCATATCAAGAGCGAGCCGT	GTTCTTGGCCCGTTGTTTCT	65
set-2	GCTCAAAGAACATGGGTGCA	GCCTATACGATGAGCCAGGT	64
set-7	CAAAGCCTATGGTCGGAAGC	GCGATTGAAGAGGAGGCTTG	66
npf	CCAAAGACAAGAGCCACACC	TGCTTGCTGGTCCCTTCATA	65
nst-1	CCGCACATTCCGCAAATCTA	ATCCTGCATAGCCATCTCCC	66
nst-2	AAAGGAACACTCGCGGACTA	GAGCACGTAGAATGGCTTGG	62
nst-4	TCGGATCTCTCTGCCATCAC	TCGGACTCTTGGGAAGCATT	67
nst-6	TCGTTCGGGTCTTTCTGTCA	TTAGGCAGAGGAAGTTGGGG	66
nst-7	CTGTAGGTGACGGTCCAAGT	AGTTGGGGAAGTTCGTTTGC	62
hda-1	AGACACTCATCCTGCCACAA	CATCGGAAATGCTCAGGGTG	64
hda-2	CGGCCTCTACGACTACTGTT	GGAAAAGATGGCCGTACTGC	62
hda-4	ACAGACACCAACTAGTCCCG	CATTGCTCCAAACACCGTCA	64
qde-1	CGGTTCCAAACGACCATCTC	ACGATGAAAGGGGCTGGTTA	66
qde-2	AGCCGAGTTCCAACCAAAAC	CCTGAAACAGCTGTGCAAGT	64
dcl-1	ATTGCCTTCTTCCTGGTCGA	CTCGGTCTTGGGTTTGCAAA	66
dcl-2	CGAGTGATGGTTGCAGATGG	CGGTCATCAGTCTGGCAATG	67
qip	CTCGCGGACATCAAGACATG	CGCGGTATCAAGAATTGCCA	67
aof2	TCCTGTGGATGCGGATAGAC	GTCCCTGGAGCTTCTTCCAT	65
elp3	CTAACCGCTATTATCGCCGC	ATGTTGCTCTGTTCTCCCGA	65
lid2	TCCCACCTCAAGCAGCATTA	GCTTTCCCTTCTGTACCCCT	64
ngf-1	CGAGAAGTACATGTGGCAGC	CTGCCATGACGACTTTCAGG	64
mat a	AGCGACTTCCTTAACACCCA	CTTGAACAGCTTGACGGGAC	64
mat A	GTCAACGGCTTCATGGGTTT	CGTTCATGGGCTGGAGATTG	64
