# Supplementary material for: Epigenetic Control of Phenotypic Plasticity in the Filamentous Fungus Neurospora crassa
Source: G3 (Bethesda). 2016 Sep 29;6(12):4009–22. doi: 10.1534/g3.116.033860 (PMC5144970; doi:10.1534/g3.116.033860)
Supplement: Supplemental Material [file supp_g3.116.033860_TableS3.pdf]

Table S3: Results of pairwise ANOVA comparing all mutants to the control in the different environments. Genotype is the effect of the genotype in the model and G×E is the effect of Genotype × Environmental setting in the model. Degrees of freedom are Genotype = 1, Environmental setting = 5, Genotype × Environmental setting = 5, Residuals = 48. Adjusted p-values have been corrected for multiple testing. A significant G×E term suggest a reaction norm shape change.

Genotype	F-value Genotype	p-value Genotype	p-adj. Genotype	F-value G×E	p-value G×E	p-adj. G×E
			Temperature environment			
aof2	38.082	1.37E-07	1.79E-06	8.163	1.23E-05	0.000197
dcl-1	2.491	0.121	1	0.328	0.893	1
dcl-2	1.008	0.32	1	0.206	0.958	1
dim-2	2.426	0.126	1	3.416	0.0101	0.111
dim-5	1500.773	7.17E-38	1.72E-36	26.766	8.08E-13	1.78E-11
dmm-1	2.178	0.146	1	0.303	0.909	1
dmm-2	30.824	1.2E-06	1.44E-05	2.042	0.0894	0.804
elp3	42.344	4.19E-08	6.29E-07	3.989	0.00417	0.0584
hda-1	186.486	3.75E-18	6.74E-17	23.080	9.9E-12	2.08E-10
hda-2	151.199	1.92E-16	3.26E-15	13.864	2.19E-08	3.72E-07
hda-4	0.359	0.552	1	1.653	0.165	0.989
lid2	41.488	5.3E-08	7.41E-07	3.934	0.00454	0.0584
ngf-1	4813.021	8.5E-50	2.13E-48	140.979	1.76E-27	4.41E-26
npf	457.147	7.38E-26	1.55E-24	17.981	6.24E-10	1.18E-08
nst-1	6.093	0.0172	0.189	3.100	0.0167	0.167
nst-2	2.354	0.132	1	0.772	0.574	1
nst-4	0.853	0.36	1	1.767	0.138	0.963
nst-6	258.363	9.99E-21	1.9E-19	19.862	1.37E-10	2.74E-09
nst-7	460.302	3.04E-26	6.68E-25	33.176	1.77E-14	4.07E-13
qde-1	0.418	0.521	1	1.899	0.113	0.903
qde-2	74.462	2.51E-11	4.01E-10	6.810	7.16E-05	0.00107
qip	3.015	0.0889	0.889	3.951	0.00442	0.0584
set-1	518.634	2.23E-27	5.13E-26	14.644	1.03E-08	1.85E-07
set-2	313.622	1.1E-22	2.19E-21	39.122	8.17E-16	1.96E-14
set-7	0.454	0.504	1	0.937	0.466	1

Continued on next page…

Table S3 – Continued

Genotype	F-value Genotype	p-value Genotype	p-adj. Genotype	F-value G×E	p-value G×E	p-adj. G×E
			Salt stress environment			
aof2	76.347	1.73E-11	3.12E-10	1.542	0.195	1
dcl-1	0.620	0.435	1	0.983	0.438	1
dcl-2	5.786	0.0201	0.18	0.505	0.771	1
dim-2	11.028	0.00172	0.0207	0.794	0.56	1
dim-5	1354.443	7.78E-37	1.79E-35	109.174	5.11E-25	1.23E-23
dmm-1	108.147	3.44E-13	6.54E-12	2.557	0.0415	0.415
dmm-2	66.000	3.76E-10	6.39E-09	4.997	0.00111	0.0133
elp3	27.116	3.96E-06	5.95E-05	14.814	8.76E-09	1.49E-07
hda-1	10.599	0.00208	0.0229	28.145	3.37E-13	6.06E-12
hda-2	15.830	0.000233	0.0031	28.311	3.04E-13	5.77E-12
hda-4	1.687	0.2	1	1.028	0.412	1
lid2	51.783	3.68E-09	5.89E-08	8.121	1.3E-05	0.000182
ngf-1	7940.876	5.63E-55	1.41E-53	440.747	7.17E-39	1.79E-37
npf	1890.438	3.28E-40	7.86E-39	46.139	3.38E-17	7.43E-16
nst-1	0.154	0.696	1	6.969	5.79E-05	0.000752
nst-2	0.446	0.508	1	0.810	0.548	1
nst-4	2.139	0.15	0.901	2.608	0.0364	0.401
nst-6	125.792	5.19E-15	1.04E-13	40.165	4.95E-16	1.04E-14
nst-7	252.470	9.5E-21	2E-19	34.139	1.05E-14	2.09E-13
qde-1	15.961	0.000221	0.0031	1.272	0.291	1
qde-2	5.053	0.0292	0.234	8.582	7.29E-06	0.000109
qip	3.497	0.0676	0.473	0.353	0.878	1
set-1	308.205	1.58E-22	3.47E-21	48.900	1.07E-17	2.47E-16
set-2	6.330	0.0153	0.153	14.548	1.13E-08	1.8E-07
set-7	0.105	0.747	1	0.177	0.97	1
			Sucrose environment			
aof2	235.953	3.71E-20	7.42E-19	1.978	0.0989	0.692
dcl-1	3.051	0.0871	0.348	3.148	0.0155	0.185
dcl-2	0.057	0.813	0.813	2.344	0.0554	0.494
dim-2	14.725	0.000363	0.00327	4.960	0.000968	0.0155
dim-5	1301.908	1.95E-36	4.28E-35	16.029	2.85E-09	5.41E-08

Continued on next page…

Table S3 – Continued

Genotype	F-value Genotype	p-value Genotype	p-adj. Genotype	F-value G×E	p-value G×E	p-adj. G×E
dmm-1	28.527	2.5E-06	3E-05	3.569	0.00798	0.104
dmm-2	177.734	9.38E-18	1.59E-16	8.640	6.78E-06	0.000115
elp3	107.831	7.27E-14	1.02E-12	4.476	0.00199	0.0279
hda-1	135.107	1.47E-15	2.35E-14	32.400	2.72E-14	6.26E-13
hda-2	207.163	4.88E-19	9.27E-18	35.049	6.43E-15	1.54E-13
hda-4	5.134	0.028	0.168	1.609	0.176	1
lid2	111.812	3.95E-14	5.92E-13	1.056	0.397	1
ngf-1	34789.772	2.51E-70	6.28E-69	208.377	2.54E-31	6.34E-30
npf	2109.907	2.49E-41	5.98E-40	2.525	0.0415	0.457
nst-1	24.917	8.29E-06	9.12E-05	0.606	0.696	1
nst-2	2.037	0.16	0.48	0.130	0.985	1
nst-4	10.674	0.00201	0.0141	2.389	0.0515	0.494
nst-6	204.769	6.13E-19	1.1E-17	12.731	6.82E-08	1.23E-06
nst-7	736.595	8.92E-31	1.87E-29	28.873	2.15E-13	4.73E-12
qde-1	12.975	0.000747	0.00598	2.416	0.0494	0.494
qde-2	1.659	0.204	0.48	4.526	0.00185	0.0277
qip	3.554	0.0655	0.327	1.031	0.41	1
set-1	2109.974	2.49E-41	5.98E-40	24.758	3.06E-12	6.43E-11
set-2	53.395	2.49E-09	3.24E-08	16.991	1.21E-09	2.42E-08
set-7	15.576	0.000258	0.00258	1.066	0.391	1
			pH environment			
aof2	53.180	2.62E-09	3.41E-08	0.223	0.951	1
dcl-1	0.999	0.323	1	0.427	0.828	1
dcl-2	0.189	0.665	1	0.835	0.531	1
dim-2	0.049	0.825	1	0.789	0.563	1
dim-5	2345.603	2.07E-42	4.76E-41	21.865	2.4E-11	5.52E-10
dmm-1	4.668	0.0357	0.357	2.037	0.0901	1
dmm-2	76.107	2.16E-11	3.46E-10	2.885	0.0237	0.403
elp3	62.737	2.9E-10	4.35E-09	3.862	0.00507	0.101
hda-1	94.304	6.56E-13	1.11E-11	5.196	0.000685	0.0151
hda-2	131.374	2.42E-15	4.35E-14	3.675	0.00677	0.129
hda-4	2.626	0.112	0.893	1.616	0.174	1

Continued on next page…

Table S3 – Continued

Genotype	F-value Genotype	p-value Genotype	p-adj. Genotype	F-value G×E	p-value G×E	p-adj. G×E
lid2	60.368	4.92E-10	6.88E-09	1.735	0.145	1
ngf-1	9005.340	2.8E-56	6.99E-55	4.667	0.00149	0.0314
npf	2376.582	1.52E-42	3.65E-41	0.715	0.616	1
nst-1	7.297	0.00952	0.105	0.644	0.667	1
nst-2	2.517	0.119	0.893	0.315	0.902	1
nst-4	0.307	0.582	1	1.107	0.369	1
nst-6	1654.564	7.38E-39	1.55E-37	94.344	1.23E-23	2.96E-22
nst-7	2334.478	2.31E-42	5.09E-41	121.382	4.94E-26	1.23E-24
qde-1	0.155	0.696	1	0.726	0.608	1
qde-2	52.449	3.13E-09	3.76E-08	3.550	0.00823	0.148
qip	3.401	0.0713	0.642	0.819	0.542	1
set-1	516.892	2.4E-27	4.8E-26	0.612	0.691	1
set-2	154.777	1.25E-16	2.37E-15	1.295	0.282	1
set-7	0.059	0.809	1	0.606	0.696	1
